# Social Support and Depressive Symptoms Among Adolescents During the COVID-19 Pandemic: The Mediating Roles of Loneliness and Meaning in Life

**DOI:** 10.3389/fpubh.2022.916898

**Published:** 2022-06-20

**Authors:** Ying Liu, Jinsheng Hu, Jia Liu

**Affiliations:** Department of Psychology, Liaoning Normal University, Dalian, China

**Keywords:** social support, loneliness, meaning in life, depressive symptoms, adolescents

## Abstract

Identifying which factors influence depressive symptom during the COVID-19 pandemic is highly significant for psychological crisis interventions among adolescents. Social support is likely to be one of the main factors. However, the underlying mechanism is still not well understood in the context of COVID-19. The current study examines whether loneliness and meaning in life mediate the association between social support and depressive symptoms in adolescents. A sample of 1,317 high school students in China were surveyed using the Perceived Social Support Scale, the Chinese Child Loneliness Scale, the Meaning in Life Questionnaire, and the Beck Depression Inventory-II. The results showed that social support predicted depressive symptoms directly and indirectly by enhancing loneliness and diminishing the sense of meaning in life. These findings help in providing new entry points in the design of effective depression prevention and intervention for adolescents during the COVID-19 pandemic.

## Introduction

The current COVID-19 pandemic has had global consequences with its high rate of infection and low predictability affecting the mental health of the public, particularly with regard to depression. Depressive symptoms have increased during the pandemic compared to periods before the outbreak (1). Moreover, most mental health problems associated with this outbreak are persistent (2). Thus, increased levels of depression has become a significant psychological crisis during the COVID-19 outbreak.

Therefore, COVID-19 crisis poses a pervasive threat to the overall health of all populations. However, the effect of the global pandemic on adolescents of great concern. Given that adolescence is a transitional and critical period in human development, poor mental health can compromise adolescents' developmental potential. However, during this period, adolescents seem especially sensitive to traumatic and stressful events, thereby leading to depression (3). For instance, a recent cross-sectional study found the prevalence of depression to be 43.7% among Chinese adolescents during the COVID-19 outbreak (4). Moreover, with the development of the pandemic, the results of two large-scale surveys has showed that the prevalence was increasing (5). In line with this, the possibility of the pandemic's continuing impact on depression suggests the importance of understanding the underlying mechanisms of pandemic-related depression in order to tailor interventions.

COVID-19 is a public health emergency that has caused great harm to the physical and mental health of the public and requires urgent risk management. As a country with a collectivism culture background, social support is a common risk management model in China. Research shows that social support plays a positive role in economic risk management, special group risk management and disease risk management (6, 7). Social support has been shown to be a protective factor for mental and physical health problems (8), helping to reduce negative emotions and the likelihood of mass incidents in public (9). COVID-19 is a national, public event with the potential for infection and death that may be beyond the scope of normal risk management. Therefore, the mechanism of the impact of social support on adolescents' mental state in the context of the pandemic needs to be explored further, which will provide reference for the pandemic-induced mental health recovery strategy.

Social support has been broadly conceptualized as the degree to which people in our social networks are responsive to our needs in the present and perceived to be responsive in the future (10). Social support includes received and perceived social support. Received social support refers to the amount of support received while perceived social support refers to its adequacy and availability (11). Research has shown that perceived social support is more closely related to mental health than received social support (12). The main-effect hypothesis argues that social support plays a role of universal gain in maintaining positive emotional experiences and mental conditions of individuals (13). Social support has been shown to be a protective factor for mental and physical health problems; lower levels of social support were more likely to lead to depressive symptoms (14). The more social support an individual receives or perceives to receive, the better they can manage sources of stress and deal with negative events. Therefore, this study proposes the following hypothesis:

Hypothesis H1: Social support is negatively associated with depressive symptoms among high school students during COVID-19.

Before the COVID-19 outbreak, researchers had used different groups to explore mediating variables between social support and depression. For example, in clinical patients, hopelessness (15), optimism (16), coping strategies (17), and self-efficacy (18) have been proved to play a mediating role between social support and depressive symptoms. In addition, self-esteem (19) and positive emotions (20) also mediated the relationship between social support and depressive symptom in adolescents. Dalian is one of the most seriously affected cities by the COVID-19 outbreak in China. It has suffered five major outbreaks since 2019. To control the outbreak, Dalian was locked down on November 9, 2021. By the time of investigation, students in Dalian have been studying at home for more than 10 days. However, due to the severity of the pandemic and the policy of home isolation, the learning environment and lifestyle of adolescents have undergone major changes, which may further aggravate the adverse effects on mental health (21). The existing mediating variables may not be able to properly explain the relationship between social support and depression based on the pandemic context.

Loneliness is the absence of imperative social relations and lack of affection in current social relationships (22). During the pandemic, governments implemented home isolation and home-schooling policies, isolating groups from previously available resources. Here, the sudden reduction in social networks may have contributed to loneliness, causing increased likelihood of loneliness among students. It should be noted that, loneliness can be reduced by social support through good social relationships. Research shows that the less support given by parents, teachers and peers, the more likely high school students are to feel lonely (23). Therefore, social support is negatively correlated with loneliness.

The Evolutionary Theory of Loneliness (ETL) suggests that social connections protect animals from hunting and resource scarcity (24). If a person becomes socially isolated, he or she will be deprived of the protection that society brings. In order to promote self-protection, individuals focus excessively on social threats. This focus bias perpetuates loneliness by creating more negative social expectations in individuals, leading to self-defeating social behaviors. In the long term, this self-reinforcing loop of loneliness may cause increased physical and mental health risks. Thus, ETL predicts that loneliness can has negative effects on physical and mental health. Therefore, in the current outbreak, loneliness may help establish a link between social support and depression. Accordingly, this research proposes the following hypothesis:

Hypothesis H2: Loneliness plays a mediating role between social support and depression symptom among high school students.

Meaning in life is defined as the strength and intensity of the efforts made by people to understand and enhance the meaning, importance, and purpose of their lives, including the presence meaning in life and search for meaning in life (25). According to Maslow's motivation theory, the most important motivation for individual action comes from the individual's most pressing needs. In the process of satisfying higher needs, individuals can experience a better sense of meaning in life (26). Therefore, from the perspective of motivation theory, the meaning in life is “continuous self-actualization.” Devogler and Ebersole (27) stated that the motivational sources of meaning in life involved environmental factors (e.g., interpersonal relationships), and individual factors (e.g., attitudes and beliefs) (27). Environmental factors are divided into socioeconomic status factors and family environment factors. As a family environment variable, social support is one of the important motivations for individuals to obtain a sense of meaning in life. Cheng and Yusooff (19) proposed that enjoyment of life, social concern, physical and mental health, harmonious relationship and self-growth are the sources of the sense of meaning in life, which also implies the important role of social support.

Scholars from different countries have pointed out that social support plays an important role in defining the meaning in life, which can be further explained by the self-determination theory (SDT). Self-determination theory holds that individuals achieve self-actualization through the integration of goals and motivations, which promotes personality perfection and mental growth (28). As a basic psychological need, the individual's search for meaning in life can affect the motivation tendency, while the perceived of meaning in life, as an embodiment of self-realization, is the result of individual's self-determination. The process of self-determination includes three parts: internal motivation, internalization of external motivation and emotional integration. The internal motivation reflects individual's interest in the behavior itself, and the internalization process of external motivation can be affected by personality factors and environmental factors such as social support. In short, social support is the external motivation necessary for deriving an individual's meaning in life. Further, positive interpersonal relationship and social support plays important roles in individuals' experience and construction of meaning in life.

In self-report studies, respondents noted that good social relationships help individuals feel valued in their life (29). Individuals with strong social support networks and positive relationships with family members and close friends felt more meaningful in life and work (30). Adolescents usually gain meaning in life from their personal experiences in relationships; they define their meaning in terms of their relationships with parents, friends, and other significant individuals (31). Furthermore, in a crisis environment of high uncertainty, maintaining a feeling of greater meaning in life can support and promote individuals' mental health, mitigating psychological harm (32). Accordingly, this research proposes the following hypothesis:

Hypothesis H3: Meaning in life plays a mediating role between social support and depressive symptoms among high school students.

As reviewed above, both loneliness and meaning in life are associated with depressive symptoms. As such, loneliness and meaning in life might influence each other and contribute to depression. In that way, we should consider two potential mediating paths. One path is that the sense of loneliness increases depressive symptoms though meaning in life. The other path is that meaning in life decreases depressive symptoms through increases in the sense of loneliness.

Theoretical and experimental evidences have showed that the former was plausible. Solitary confinement can increase psychiatric symptoms of prisoners (33). Accordingly, deconstruction hypothesis proposed that loneliness simulates the impotence and worthlessness of death (34). Moreover, loneliness may lead to individual's state of deconstruction, they will only focus on specific real-time events and deal with their own situation from a narrow perspective. The hypothesis holds that meaningful thought is an important basis for self-awareness and emotion. Individuals in deconstruction state are prone to this kind of trouble, while lonely individuals will be in a state of mind that life is meaningless. In other words, deconstruction hypothesis predicted that loneliness may lead to a thought that meaningless in life.

A series of experiments have validated the predictions of deconstruction hypothesis. Dissatisfaction with the quality or quantity of social connections can lead to loneliness (35). Klein (36) used a spent money task to test the determinant of meaning in life. When controlled for participants' happiness after they spent money on other people, the result showed that meaning in life was positively correlated with the social connection to others, suggesting a correlation between loneliness and meaning in life. Stillman et al. (37) evaluated the relationship between loneliness and meaning in life by three different questionnaires and experiments. Four studies included laboratory-administered and naturally-administered loneliness experiences. The results showed that loneliness was associated with lower sense of life meaning. The experimental design of experiments 1 and 2 allows causal inference; therefore, it can be concluded that loneliness is the direct cause of a reduced sense of meaning in life. Accordingly, this research proposes the following hypothesis:

Hypothesis H4: Loneliness and meaning in life mediate the relationship between social support and depressive symptoms among high school students through their chain-mediating effect.

AS a common risk management model, social support plays an essential role in mental health during pandemic. However, few studies have explored the serial mediating mechanism of “social support-depressive symptoms” during home-schooling in the COVID-19 outbreak period for high school students. Particularly, there is a lack of study of the relationship between social support, loneliness, meaning in life, and depressive symptoms. Based on the current literature, this study observes the relationship between social support and depressive symptoms and the mediating effect of loneliness and meaning in life.

The findings will provide ideas for the improvement of students' mental health during the COVID-19 outbreak. The ultimate goal of this study was to improve the effectiveness of social support interventions through reducing sense of loneliness and increasing the sense of meaning in life.

## Methods

### Participants and Procedure

The survey began on November 19, 2021, and ended on November 20, 2021 during COVID-19 outbreak at a high school. The pandemic in Dalian began on November 4, and as of November 19, there were 285 confirmed COVID-19 cases and 36 asymptomatic infected persons in two high-risk areas and 45 medium-risk areas. Since November 9, the government has ordered all students to take online classes at home, while residents are not allowed to go out at will and leisure activities are restricted. The homeschooling style was the combination of live or recorded broadcasts and communication by WeChat or other social software.

The Ethics Committee of the Department of Psychology at Liaoning Normal University approved this study. After obtained the approval and cooperation of the teaching department of the high school, we asked the teacher in charge of classes to share the questionnaire link to the WeChat groups of the students' parents. The online questionnaire was hosted by Wenjuanxing (https://www.wjx.cn/). If the parents agree to their children answering the questionnaire, after replying “agree” in the group, the child will use the parents' mobile phone to fill in their responses. However, the questionnaire was collected anonymously. Children who did not obtain parental consent could not click the link to answer the questionnaire. On the front page of the online questionnaire, we explained the research intention to the students and emphasized the principle of voluntary, anonymous and truthful answers. They could click on the start button to automatically jump to the formal survey questions. Participants were free to withdraw from the survey at any time in 2 days.

The survey data showed that all parents at the school agreed to their children filling out the questionnaire. A total of 1,387 high school students were recruited online. After eliminating questionnaires that consistently selected the same options, 1,317 (mean age = 15.96 years, SD = 0.81, range = 15–17 years old) questionnaires were analyzed. Effective recovery rate is 94.9%. The sample characteristics are shown in Table 1. Although we did not calculate sample size before data collecting, we have found enough samples to explore the research question, which should be effective. In addition, we did not register the hypotheses and analysis plan before collecting data; therefore, we do not have any preregistration.

**Table 1:** Sample background characteristics.

		***N* (%)**
Gender	Male	609 (46.24%)
	Female	708 (53.76%)
Grade	Senior one	460 (34.93%)
	Senior two	450 (34.17%)
	Senior three	407 (30.90%)
Residential area	Rural areas	128 (9.72%)
	Urban areas	1,189 (90.28%)

### Materials and Measures

#### The Perceived Social Support Scale

The perceived social support scale (PSSS) was developed by Zimet et al. (38) and revised by Jiang (39), a Chinese researcher. The Cronbach's alpha coefficients of the PSSS was 0.83 among Chinese students. Confirmatory factor analysis indicated that χ^2^/df = 2.09, IFI = 0.95, CFI = 0.95, RMSEA = 0.05 (40). PSSS includes 12 items to assess perceived support arising from three groups, namely family, friends, and significant others. Items were rated on a 7-point scale ranging from 1 (extremely disagree) to 7 (extremely agree), greater score indicating a higher level of perceived social support. Cronbach's alphas were 0.95 in the present study.

#### The Child Loneliness Scale

The Child Loneliness Scale (CLS) was developed by Asher et al. (41) and was revised into Chinese by Li et al. (42). Compared with the original scale, several items of the Chinese CLS were reword to fit adolescents in China. The Chinese CLS consisted of 21 items and four subscales: loneliness (e.g., “I have lots of friends at school”), feelings of social adequacy vs. inadequacy (e.g., “I'm good at working with other children”), subjective estimations of peer status (e.g., “My classmates like me”), social dissatisfaction (e.g., “It's hard for me to let other classmates to like me”). Participants were asked to indicate how much each statement was a true description of themselves. The items were rated on a 5-point Likert scale ranging from 1 (not true at all) to 5 (always true), greater score indicates a higher level of loneliness. The modified scale has also been found to have good reliability and construct validity in the sample of high school students (42). Cronbach's alpha for the total scale was 0.74.

#### The Meaning in Life Questionnaire

The Meaning in Life Questionnaire (MLQ) was compiled by Steger et al. (43) and was revised by Wang (44). The Chinese revision of the MLQ was found to have good internal consistency, construct-convergent validity and discriminant validity in samples of Chinese adolescents (44). The MLQ consisted of 10 items and two subscales: the presence of meaning and the search for meaning. Participants respond to the items on a 7-point scale ranging from 1 (“absolutely untrue”) to 7 (“absolutely true”). Higher scores indicating higher presence and search. In the current study, Cronbach's alphas for the total scale were 0.76.

#### Beck Depression Inventory-II

Depression symptoms was assessed by the Chinese version of Beck Depression Inventory-II (45, 46), a 21 items self-report measure of depressive symptoms over the past 2 weeks. Each item is rated on a 4-point Likert scale, ranging from 0 (absence) to 3 (severe presence). The Cronbach's alpha coefficients of the BDI-II-C was 0.89, and the test-retest reliability was 0.93 among adolescents of China. Confirmatory factor analysis indicated that χ^2^/df = 2.87, IFI = 0.96, CFI = 0.97, RMSEA = 0.026 (46). Summed to derive a total score, greater scores indicating higher levels of depression symptoms. Cronbach's alpha for the total scale was 0.92.

### Statistical Analysis

Data analysis was conducted using SPSS Statistics 19.0. Descriptive statistics were computed for all sociodemographic information available and all study variables. We used Chi-square test to examine group differences in terms of gender, grades and residential area. The PROCESS macro program was used for the mediation analysis, repeated sampling 5,000 times from the original data to calculate the 95% CI. If the 95% CI of the standardized path coefficient does not contain 0, it indicates that the mediating effect is significant. By logistic regression analysis, we test the predictive effect of the model on low and high depressive symptom scores.

## Results

### Test of Validity and Common Method Bias

In this study, we only collected data with self-reporting method and common method bias (CMV) may occur (47). To further improve the rigor of the study, we used Harman's single-factor test to test common method deviations before data analysis. The results showed that there were 9 factors with eigenvalues >1, which explained 62.75% of the variation, and the variation explained by the first factor was 34.45%, which was less than the critical value of 40% (47). Therefore, there is no serious common method biases in this study.

### Preliminary Analysis

One thousand three hundred and seventeen high school students fulfilled the entry criteria of this study. The BDI-II mean score was 6.47 (SD = 8.03), 16.25% suffered from depression. Among the 214 students with depression, 50% (107/214) reported mild depression, 33.64% (72/214) moderate depression, and 15.42% (33/214) severe depression. The differences between control variables has no significance, such as gender, χ^2^ (1, *N* = 1,317) = 1.421, *p* = 0.233, grades, χ^2^ (2, *N* = 1,317) = 2.837, *p* = 0.242, and residential area, χ^2^ (1, *N* = 1,317) = 0.003, *p* = 0.960.

### Correlations

Partial correlations for both samples are reported in Table 2. Depressive symptoms was negatively correlated with social support and meaning in life and was positively correlated with loneliness. Social support was negatively correlated with loneliness and was positively correlated with meaning in life. Finally, meaning in life was negatively correlated with loneliness. The significant correlation between research variables provides a good foundation for subsequent research hypotheses and mediation testing.

**Table 2:** Bivariate correlations between variables of interest.

**Variables**	***M***	***SD***	**1**	**2**	**3**	**4**
1 Depressive symptoms	6.47	8.03	1.00			
2 Social support	68.45	12.56	−0.55***	0.56***	1.00	
3 Meaning in life	53.29	10.46	−0.44***	−0.71***	−0.49***	
4 Loneliness	39.96	15.16	0.55***	0.56***	1.00	1.00

***p <0.001.

### Testing for the Mediation Effects

Based on the results of the correlation analysis and our hypothesis that loneliness and meaning in life mediate the relationship between social support and depressive symptoms, we used PROCESS Model 6 to test the mediating model. The scores on all variables in the path analysis were converted to z-scores. The first regression analysis tests the effects of social support on loneliness (path a1). The second regression model tests the combined predictive effects of social support and loneliness on meaning in life (paths a2 and d). The third regression predicts the depressive symptom by the independent variable social support and the two mediators (paths b1, b2, and c′). Here, path c′ depicts the direct effect of social support on the depressive symptom controlled for the effects of the two mediators. In contrast, path c indicates the total effect of social support on depressive symptom without considering the mediators.

Model indices are depicted in Table 3. First, in the path of a1 → b1, social support had a significant negative effect on loneliness (β = −0.711, *p* < 0.001), while loneliness had a significant positive effect on depressive symptom (β = 0.296, *p* < 0.001). In the path of a2 → b2, social support had a significant positive effect on meaning in life (β = 0.440, *p* < 0.001), while meaning in life had a significant negative effect on depressive symptom (β = −0.153, *p* < 0.001). In the path of a1 → d → b2, loneliness had a significant positive effect on meaning in life (β = −0.172, *p* < 0.001). These results supported hypotheses 1–4.

**Table 3:** Regression analysis results.

**Model**	**Outcome**	**Predictors**	**β**	***SE***	***t***	**LLCI**	**ULCI**
Model 1	Loneliness	SS	−0.711	0.021	−34.537***	−0.751	−0.671
	*R^2^*= 0.505, *F* = 1192.797***						
Model 2	MIL	SS	0.440	0.035	12.436***	0.371	0.510
		Loneliness	−0.172	0.034	−5.048***	−0.239	−0.105
	*R^2^*= 0.331, *F* = 280.727***						
Model 3	DS	SS	−0.248	0.039	−6.289***	−0.325	−0.171
		Loneliness	0.296	0.035	8.550***	0.228	0.364
		MIL	−0.153	0.031	−4.948***	−0.214	−0.092
	*R^2^*= 0.364, *F* = 158.865***						

MIL, Meaning in life; SS, Social support; DS, Depressive symptom; LLCI, Boot CI lower limit; ULCI, Boot CI upper limit. The *** symbol indicates the value of p <0.001.

For the prediction of depressive symptom (Table 4), social support was a statistically significant and negative predictor (*effect of c* = −0.545, *p* < 0.001) in the total effect model without consideration of the mediators. However, the explained variance increased by Δ*R*^2^ = 0.141 when the mediators, loneliness and meaning in life, were included in the model. The direct effect was reduced by inclusion of the mediators but remained significant [*effect of c*′ = −0.248, *p* < 0.001; 95% CI (−0.325, −0.171)], whereas the total indirect effect was significant [*total indirect effect* = −0.297, *p* < 0.001; 95% CI (−0.359, −0.239)]. Correspondingly, all three possible indirect effects were significant [*effect of a1*→*b1:* = −0.210, 95% CI (−0.263, −0.161); *effect of a2*→*b2:* = −0.067, 95% CI (−0.099, −0.041); *effect of a1*→*d*→*b2* = −0.019, 95% CI (−0.032, −0.010)].

**Table 4:** Total, direct and indirect effects of social support on depression.

**Model**	**Effect**	**SE**	**LLCI**	**ULCI**	**Ratio**
Total effect (c)	−0.545	0.029	−0.601	−0.488	100%
Direct effects (c′)	−0.248	0.039	−0.325	−0.171	45.50%
Total indirect effect	−0.297	0.030	−0.359	−0.239	54.50%
SS → Loneliness → DS (a1 → b1)	−0.210	0.026	−0.263	−0.161	38.53%
SS → MIL → DS (a2 → b2)	−0.067	0.015	−0.099	−0.041	12.29%
SS → Loneliness → MIF → DS (a1 → d → b2)	−0.019	0.006	−0.032	−0.010	3.49%

**Figure 1:**
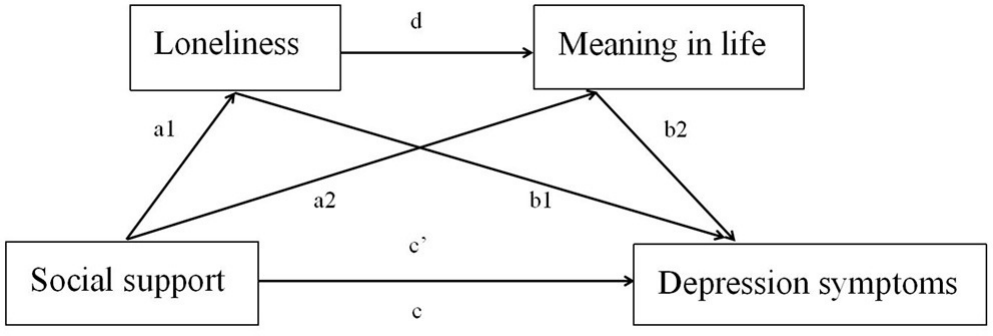
Roadmap of the influence of social support on depressive symptoms.

Those findings indicated that loneliness and meaning in life mediated the association between social support and depressive symptoms, respectively, through the chain intermediary of loneliness and meaning in life. The final model for the whole sample is shown in Figure 1.

### Logistic Regression Analysis

The model created included three independent or predictor variables of social support (X1), loneliness (X2), and meaning in life (X3), and one dependent or criterion variable of low depressive symptom score (BDI ≤ 13) and high depressive symptom score (BDI > 13). Social support, loneliness and sense of meaning in life were all entered in the regression equation (*p* < 0.001) (see Table 5). The regression equation was Logit (*P*) = −0.032X1 + 0.054X2 – 0.046X3. The test of the likelihood of regression equation was significant, χ^2^ = 295.001, df = 3, *p* < 0.001. It indicates that when using these variables of social support, loneliness and meaning in life, the estimated model has better fitness for the sample than the null model (when the coefficients of all variables are 0). The Wald test result showed that the three independent variables were significant (*p* < 0.001), indicating certain explanatory ability to the model.

**Table 5:** Variables in the regression equation.

	***B***	**SE**	**Wald χ^2^**	**df**.	**Sig**.	**Exp (*B*)**	**95% Confidence intervals for EXP(B)**	
							**Lower**	**Upper**
X1	−0.032	0.009	11.860	1	0.001	0.969	0.951	0.986
X2	0.054	0.008	51.283	1	<0.001	1.056	1.040	1.071
X3	−0.046	0.010	21.066	1	<0.001	0.955	0.937	0.974
Constant	0.234	0.871	0.072	1	0.788	1.263		

*X1, Social support; X2, Loneliness; X3, Meaning in life*.

In the low depressive symptom score group, 96.6% were correctly predicted; in the high depressive symptom score group, 30.4% were correctly predicted. Together these three variables accounted for 85.5% of the variance in low/high depressive symptom score individuals.

## Discussion

The current study had high school students as participants and aimed to investigate whether social support has effect on mental health in the context of pandemic. In addition, we also investigated whether adolescents' loneliness and meaning in life mediated the association between social support and depressive symptoms. We found one direct effect and three indirect effects: (1) social support → depressive symptoms, (2) social support → loneliness → depressive symptoms, (3) social support → meaning in life → depressive symptoms, and (4) social support → loneliness → meaning in life → depressive symptoms.

### Relationship Between Social Support and Depressive Symptom

This study found that social support had a direct negative effect on the depressive symptoms of adolescents, which is consistent with Liu et al. (48) and Marroquín et al. (49). The results of the current study supported and verified the main-effect model of social support and supported the view that “social support may mitigate the psychological consequences of social lockdown during the spread of COVID-19” (50).

Individuals who perceived a high level of social support experience reduced occurrences of emotional and behavioral problems which can directly alleviate adolescents' depressive symptoms (51, 52). Thus, high levels of perceived social support are conducive to adolescents coping well with the many pressures resulting from the COVID-19 outbreak. Particularly, they experienced enhanced confidence in solving difficulties and problems during the study-at-home period. In other words, low levels of perceived social support will negatively impact adolescents' mental health and may lead to adolescents' depression. In the end, perceived social support can help to reduce the incidence of adolescents' depressive symptoms during the COVID-19 pandemic. Thus, hypothesis H1 is supported.

### Loneliness' Role Between Social Support and Depressive Symptoms in Adolescents

Results showed that social support had indirect effects on depressive symptoms through the mediating effect of loneliness. Loneliness has an inverse relationship to the number of family and friends and how much support an individual perceives to receive from them (53). Previous studies have also reported that providing social support was a core type of loneliness intervention (54). However, stay-at-home policies during the COVID-19 pandemic led to the sudden reduction of social networks among adolescents, thereby impacting students' access to social support. Additionally, stay-at-home policies also indicated significant decline in the enjoyment of social leisure activities as adolescents lost an important way to maintain intimate relationships (55).

Without close confidants and supportive social networks, adolescents might be faced with loneliness; so, an increased sense of loneliness may increase the risk of depression (56). A study demonstrated that lonely students were six times more likely to be affected by depressive symptoms (57). It is not difficult to conclude that loneliness plays a “bridging” role between social support and depressive symptoms through previous research supported by our results. Thus, hypothesis H2 is supported.

### Meaning in Life' Role Between Social Support and Depressive Symptoms in Adolescents

The present results showed that meaning in life also played a mediating role between social support and depressive symptoms. This finding was in accordance with the existing literature. For example, a questionnaire survey explored the relationship between meaning in life and depressive symptoms in young men; the results showed that meaning in life is an important discriminative factor among lower, medium, and higher depressive symptoms. Here, the higher the meaning in life, the fewer depressive symptoms the participants had (58).

Furthermore, meaning in life refers to how people construct their daily experience rather than a general and unspecific type of meaning. This kind of daily sense of meaning in life can change in response to positive or ordinary life events; moreover, good social relations can contribute to the construction of meaning in life. In an experimental study, social relations were manipulated through instructions; participants were divided into a remembered, forgotten, complimented, or control group (59). The results suggested that no lasting personal bond led to a lowered sense of meaning in life. Therefore, as one aspect of social relationships, social support has been confirmed to be positively related to meaning in life (60). According to our results, hypothesis H3 is clearly established.

### Loneliness and Meaning in Life' Role Between Social Support and Depressive Symptoms in Adolescents

Previous studies have confirmed the relationship between loneliness and meaning in life (61). The present study found that the two variables had a chain mediating effect in the process of social support affecting depressive symptoms, constituting an intermediate link in the influence path of social support → loneliness → meaning in life → depressive symptoms. This result suggests that loneliness and meaning in life not only mediated the relationship between social support and depressive symptoms independently but also affected depressive symptoms indirectly through loneliness.

In this case, loneliness is an important mediating variable of chain mediation. The Loneliness Model posits that individuals feel insecure when they do not feel social support and this sets off unconscious surveillance for social threats, eventually leading to cognitive biases (62). That is to say, lonely individuals perceive society as threatening and this results in negative social interactions in which they may enforce distancing from potential partners and attribute poor social connections to others. This vicious cycle of loneliness is accompanied by stress, hostility, and pessimism and can affect an individual's emotional and cognitive processes and outcomes.

Combined with Park's meaning-making model in a pressure context, the chain mediating effect of life meaning can be deeply explored. The meaning-making model proposes that, in the context of stress, individuals seek meaning in life to reduce the gap between situational meaning and global meaning and recover the meaning in their own life as much as possible (63). For example, economically disadvantaged individuals (situational meaning) may seek meaning in life by focusing on good personal achievement (global meaning); so, searching for meaning in life can benefit adolescents' development (64). Individuals with a strong sense of meaning in life actively promote situational meaning to assimilate with global meaning, while individuals with a weak sense of meaning in life reduce global meaning to accommodate situational meaning.

In sum, the mediating mechanisms played by loneliness and meaning in life between social support and depression are summarized as follows. The COVID-19 pandemic had led to adolescents feeling less social support and increasing loneliness. When this happens, individuals with weak senses of meaning in life blame those who provide them with social support, while constantly reducing contact with the outside world to maintain their expectations, ultimately leading to an increase in depressive symptoms. While, individuals with a high sense of life meaning adjusted their emotions when they were lonely and actively faced the inconvenience caused by the pandemic. The sense of significance and purpose kept them in the process of self- enhancement, which benefits mental health and reduces depressive symptoms. Thus, hypothesis H4 is supported.

### Limitations and Future Research

This study had several limitations. First, it used a sample of high school students during the COVID-19 outbreak, which may limit the generalizability of the findings to other populations. While the whole nation is under pressure due the pandemic, the results may not apply to adolescents in cities without outbreaks. A wider and more diverse sample may help to circumscribe the possible impact of the specific sample of this study. Second, the data was collected online and *via* self-report questionnaires; the use of a self-reported survey may be subject to social desirability bias. Furthermore, data on the psychiatric diagnoses of the participants could not be ascertained. Third, the use of the cross-sectional studies makes it impossible to infer the causal relationship between variables; future studies should extend our study using follow-up design and experimental studies.

Moreover, the participants were all from the same nation which may result in the ignorance of cultural differences in variables. Interestingly, residents of developing contexts have shown to report more meaning in life than those of developed ones (65). In a study of American college students, meaning in life did not mediate the relationship between loneliness and depressive symptoms (66). The researchers proposed that the loneliness may be a more significant factor in the influence of depression than meaning in life. Thus, in the future, the importance between meaning in social relationships and other domains of life could be compared among different cultures.

In addition, the depressive symptom scores indicate that a floor effect has occurred. The result of logical regression showed that low depressive symptom scores have little effect on the results. We analyzed three possible reasons for the floor effect in depressive symptom scores in the present study. First, this may be related to the choice of measurement scale. A meta-analyze research involved 49,656 Chinese participants revealed that the prevalence of depression during COVID-19 outbreak was 26.9% (67). The studies in the meta-analysis used a variety of depression scales, which may differ in prevalence diagnosis due to their classification criteria for depression. For example, Patient Health Questionnaire-9 (PHQ-9) is more directly reflects DSM-IV severe depressive episode criteria (68). Thus, the analysis of five studies found the prevalence of depression was 35.5%. Similarly, the analysis result of Self-rating Depression Scale (SAS) revealed that the depression prevalence was 34.1% in Chinese people. The alterations in depression prevalence ranged from 0.2 to 1.9 as a result of the analysis method, thus, the depression prevalence by BDI-II may be 25% or lower (69, 70). Second, this high school has paid more attention to mental health education. During the period of home isolation, qualified mental health teachers guided students through courses. Third, despite living in home isolation, the basic livelihood of the residents has been secured and depression may be alleviated due to the favorable supervision and rich experience of the state and government.

The present research aimed at exploring that as a risk management model, whether social support can decline depressive symptoms among high school students in the context of COVID-19 outbreaks. This aim confirmed the role of social support as an independent variable in the mediation model. In fact, social support may play as mediating or moderating role in the present model, which has been confirmed in the elderly. Liu et al. (71) found that social support mediates loneliness and depression in elderly. In individuals approaching retirement, social support had moderation effect between meaning in life and mental health (72). Multiple mediation models can be obtained by using the same variable. When constructing the mediation model, the research purpose and hypothesis should be clarified at first. This also inspires us that in order to obtain multidimensional depression intervention in the context of pandemic, we can use a variety of independent variables in future studies to explore the mediation model with depressive symptoms as the dependent variable. In addition, the mediation model in the present study also should be further explained and explored theoretically.

Despite these limitations, the current study considerably extends our understanding of the underlying mechanisms playing roles between social support and depressive symptom in adolescents during the COVID-19 pandemic. Based on the findings, individuals with more social support might have fewer depressive symptoms. Moreover, the current study reveals the mediating role of loneliness and meaning in life in the association between social support and depressive symptoms. The significant path from social support through loneliness and meaning in life to depressive symptoms further sheds light on the complex relationships among these variables. Considering the probable mechanisms, loneliness improvement programs may have a preventive function if implemented through developing social skills, increasing opportunities for social interaction, or recognizing maladaptive social cognition among adolescents (73).

Additionally, the results provide critical evidence for understanding that meaning in life may help adolescents put the COVID-19 pandemic into perspective and reduce attention to such social threats. The exploration of adolescents' senses of meaning in life is determined by the important factors that influence adolescents' depressive symptoms. Further studies should pay attention to the potential role of good relationships and personal growth in the development of meaning in life. Future studies should also try to examine the effect of different aspects of social support on adolescents, such as family function, friendship, and other social networks.

The purpose of this study is to explore the effect of social support as a risk management method on the mental health of high school students in the context of pandemic. We also explored the mechanism between social support and depressive symptoms. This study finds that adolescents with higher levels of social support have fewer depressive symptoms than those with lower levels of social support. Loneliness, meaning in life, and the combination of the two are established as mediators of social support and depressive symptoms in adolescents. The confirmation of the path related to social support and depressive symptoms provides a reference for the mental health interventions among adolescents during COVID-19 outbreaks.

## Data Availability Statement

The raw data supporting the conclusions of this article will be made available by the authors, without undue reservation.

## Ethics Statement

The studies involving human participants were reviewed and approved by the Ethics Committee of the Department of Psychology at Liaoning Normal University. Written informed consent to participate in this study was provided by the participants' legal guardian/next of kin. Written informed consent was obtained from the individual(s), and minor(s)' legal guardian/next of kin, for the publication of any potentially identifiable images or data included in this article.

## Author Contributions

YL and JH contributed to the conceptual conception of the manuscript. YL have written the draft of the manuscript. YL, JH, and JL provided important intellectual input at all stages and reviewed and revised the manuscript. Both authors approved it for publication. All authors contributed to the article and approved the submitted version.

## Funding

This research was funded by the National Social Science Fund of China, Grant Number: BIA200182.

## Conflict of Interest

The authors declare that the research was conducted in the absence of any commercial or financial relationships that could be construed as a potential conflict of interest.

## Publisher's Note

All claims expressed in this article are solely those of the authors and do not necessarily represent those of their affiliated organizations, or those of the publisher, the editors and the reviewers. Any product that may be evaluated in this article, or claim that may be made by its manufacturer, is not guaranteed or endorsed by the publisher.
